# Graph Analysis of Verbal Fluency Tests in Schizophrenia and Bipolar Disorder

**DOI:** 10.3390/brainsci12020166

**Published:** 2022-01-27

**Authors:** Adrian Andrzej Chrobak, Aleksander Turek, Karolina Machalska, Aleksandra Arciszewska-Leszczuk, Anna Starowicz-Filip, Anna Julia Krupa, Dominika Dudek, Marcin Siwek

**Affiliations:** 1 Department of Adult Psychiatry, Jagiellonian University Medical College, Kopernika St. 21a, 31-501 Kraków, Poland; adrian.chrobak@uj.edu.pl (A.A.C.); dominika.dudek@uj.edu.pl (D.D.); 2 Student’s Scientific Association of Affective Disorders, Jagiellonian University Medical College, Kopernika St. 21a, 31-501 Kraków, Poland; alekt94@gmail.com (A.T.); karolamachalska@gmail.com (K.M.); 3 Faculty of Psychology, SWPS University of Social Sciences and Humanities, Polna St. 16/20, 81-745 Sopot, Poland; aarciszewska@swps.edu.pl; 4 Department of Medical Psychology, Jagiellonian University Medical College, Jakubowskiego St. 2, 30-688 Kraków, Poland; starow@poczta.onet.pl; 5 Department of Neurosurgery, University Hospital in Krakow, Jakubowskiego St. 2, 30-688 Kraków, Poland; 6 Department of Psychiatry, Jagiellonian University Medical College, Kopernika St. 21a, 31-501 Kraków, Poland; annajuliakrupa@doctoral.uj.edu.pl; 7 Department of Affective Disorders, Jagiellonian University Medical College, Kopernika St. 21a, 31-501 Kraków, Poland

**Keywords:** speech graphs, semantic network, cognitive impairments

## Abstract

Verbal Fluency Tests (VFT) are one of the most common neuropsychological tasks used in bipolar disorder (BD) and schizophrenia (SZ) research. Recently, a new VFT analysis method based on graph theory was developed. Interpreting spoken words as nodes and every temporal connection between consecutive words as edges, researchers created graph structures, allowing the extraction of more data from participants’ speech, called Speech Graph Attributes (SGA). The aim of our study was to compare speech graphs, derived from Phonemic and Semantic VFT, between SZ, BD, and healthy controls (HC). Twenty-nine SZ patients, twenty-nine BD patients, and twenty-nine HC performed Semantic and Phonemic VFT. Standard measures (SM) and 13 SGA were analyzed. SZ patients’ Semantic VFT graphs showed lower total word count and correct responses. Their graphs presented less nodes and edges, higher density, smaller diameter, average shortest path (ASP), and largest strongly connected component than the HC group. SM did not differentiate BD and HC groups, and patients’ Semantic VFT graphs presented smaller diameter and ASP than HC. None of the parameters differentiated BD and SZ patients. Our results encourage the use of speech graph analysis, as it reveals verbal fluency alterations that remained unnoticed in the routine comparisons of groups with the use SM.

## 1. Introduction

Schizophrenia (SZ) and bipolar disorder (BD) are severe mental disorders that share common symptom dimensions, neurophysiology, and genetics, and their treatment strategies are similar [1,2,3]. Both disorders are characterized by cognitive dysfunctions involving alteration of the structure of language. Verbal Fluency Tests (VFT) are one of the most common neuropsychological tasks used in BD and SZ research. These tests evaluate the ability to produce a correct sequence of spoken words during a limited time interval [4,5]. During VFT, participants are asked to name as many words as possible starting with a specific letter (Phonemic VFT), or belonging to a specific category, e.g., animals (Semantic VFT). Those tasks deliver information about the integrity of lexico-semantic memory and the ability to recall items from it, self-monitoring, inhibition of responses in adequate situations, and effortful self-initiation [6,7].

Studies indicate that BD and SZ patients present deficits during VFT [8]. Among SZ, deterioration in semantic fluency is more common and severe than in the phonemic one [9,10]. A meta-analysis has shown that semantic VFT performance varies along the SZ continuum [11]. Patients with recent-onset psychosis, chronic SZ, as well as first degree relatives present a significantly lower number of correct responses than HC. Moreover, patients with chronic SZ show more non-perseverative errors [11]. In the case of BD, a meta-analysis revealed that patients present verbal fluency deficits with a medium effect size [12]. Unlike in SZ, there was no significant difference between semantic and phonemic VFT. Also, there was no significant effect of mood state when examined across tasks; however, in the case of semantic VFT, there is significant difference in effect size, indicating greater impairment in euthymic patients [12]. The first quantitative review comparing those two disorders showed that BD patients outperform the SZ group in both Phonemic and Semantic VFT [13]. However, a further systematic review indicated comparable neurocognitive impairments in both groups [14]. The majority of recent studies show no differences between SZ and BD [15,16,17,18], though one shows milder verbal fluency deficits in BD than in SZ [19].

Generally, VFT are being interpreted using only a number of spoken words, e.g., word count, number of correct words (productivity score), repetitions (perseverative errors), and non-perseverative errors [11]. Results being only a single characteristic are an important limitation of a neuropsychological test in clinical practice and research. As a consequence of this, many authors have considered addition aspects of VFT in their studies, adopting a qualitative approach for better understanding of the organization of semantic memory. One of these are clustering and switching scores [11]. These variables were introduced after the observation that, during VFT, respondents generate a sequence of words that can be grouped into semantic subcategories called clusters, and change from one subcategory to another, which are called switches [11,20]. It has been shown that SZ patients demonstrated a smaller cluster size and fewer instances of switching than HCs [11]. The observed deficits may represent degraded semantic store with less category exemplars available. It has been suggested that such impairments may be storage-related [11,21,22,23]. A study using cluster analysis has shown that BD patients’ strategies for categorization in semantic memory may be less related their knowledge compared to HC [24]. Patients present a reduced number and aberrant clustering of produced words. Sung et al., 2013, showed less coherent clustering of semantic exemplars in BD patients [25]. It has been proposed that those deficits may indicate impaired semantic activation/inhibition, insufficient spreading across the semantic network, or impairment regarding the control of these functions [12,25].

In recent years, Mota et al., 2012, developed an innovative method of speech analysis using mathematical graph theory [26]. Interpreting spoken words as nodes, and every temporal connection between consecutive words as edges, researchers created graph structures, allowing the extraction of more data from participants’ speech. Mota et al., 2014., delivered a software called SpeechGraphs, which generates graph models with specific Speech Graph Attributes (SGA) from a set of written words, e.g., its density, diameter, and characteristics of loops between repeated words [27]. Transforming spoken words into a network enables an analysis of their hidden features, which can help understand the dynamics and organization of cognitive processes [5]. This approach was successful in differentiating SZ and BD patient groups through analysis of participants’ dreams reports [27], or transcribed interviews with the patients [26]. Graph analysis of VFT was used only in the single study that showed that this method successfully differentiated groups of patients with Alzheimer’s Disease, mild cognitive impairment, and healthy controls (HC) [5]. This study inspired us to use this approach in the group of SZ and BD patients.

The aim of our study is to compare the properties of speech graphs, derived from Phonemic and Semantic VFT, between SZ, BD, and HC. We hypothesize that SGA can differentiate the abovementioned groups. In opposite to the correct speech graph described by Bertola et al., 2014 [5], we hypothesize that, due to the suspected higher number of errors and repetitions and lower word count, BD and SZ patients will produce non-linear networks. Compared to HC, patients should present lower numbers of nodes, words, and edges; indicators of recurrence (i.e., presence of parallel edges, repeated edges or loops); and the presence of strongly connected components. Patients’ graphs should present higher density, increased clustering coefficient (CC), and large distances. We also hypothesize that BD and SZ will present more profound deficits in Semantic than in Phonemic VFT measures.

## 2. Materials and Methods

### 2.1. Participants

Eighty-seven participants were recruited to this study: twenty-nine BD patients, twenty-nine SZ patients, and twenty-nine HC. All groups were matched in terms of age and gender (Table 1). A consensus diagnosis was made by two experienced psychiatrists according to DSM-5 and ICD-10 criteria. Inclusion criteria for patients were symptomatic remission (PANSS score of 3 or less, on all of its items), and treatment with antipsychotic drugs from the group of dibenzoxazepines (olanzapine, quetiapine, clozapine). BD patients were in euthymia, classified as <11 points in the Montgomery–Asberg Depression Rating Scale [28], and <5 points in the Young Rating Scale for Mania [29]. We selected patients treated with the antipsychotics from the dibenzoxazepine group to provide a relative pharmacological homogeneity across patient groups. In the case of BD patients, the use of lamotrigine and valproic acid was also accepted. Exclusion criteria involved history of alcohol or drug abuse according to substance use disorder of DSM-5; severe, acute, or chronic neurological and somatic diseases; and treatment different than that mentioned above. HC consisted of mentally healthy volunteers recruited from researchers’ social network. This group did not meet any of the exclusion criteria for patients. All participants signed an informed written consent prior to the assessment. The study was approved by the Bioethics Committee of the Jagiellonian University Medical College in Cracow.

### 2.2. Fluency Tests

Semantic and Phonemic VFTs were used. During Semantic VFT, participants were asked to produce the maximum number of words from a category of animal without repetitions in one minute. During Phonemic VFT, the category of words beginning with the letter “K” was used, with the same instructions as above. Word sequences were recorded and transcribed. The following standard measures were calculated separately for Semantic and Phonemic VFT: word count, total number of correct words, total number of errors, and total number of repetitions.

### 2.3. Graph Analysis

**Figure 1:**
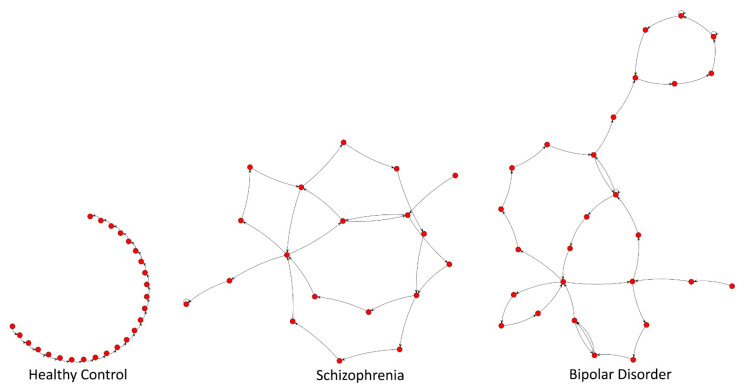
Examples of speech graphs generated from Semantic Verbal Fluency Test results for healthy control, schizophrenia, and bipolar patients.

Transcribed word sequences from Semantic and Phonemic VFT were analyzed with the use of SpeechGraphs software [27]. This tool represented words as speech graphs—every word in a sequence was presented as a node (N), and the temporal link between words as an edge (E) (Figure 1).

Thirteen Speech Graph Attributes (SGA) variables were extracted. The definitions of the following variables were derived directly from Bertola et al., 2014 [5]:

Connected components:

- “the largest strongly connected component (LSC): number of nodes in the maximal subgraph in which all pairs of nodes are reachable from one another in the directed subgraph (node a reaches node b, and b reaches a).”

Recurrence attributes:

- “repeated (RE): sum of all edges linking the same pair of nodes,
- parallel edges (PE); sum of all parallel edges linking the same pair of nodes given that the source node of an edge could be the target node of the parallel edge,
- cycles of one node (L1): sum of all edges linking a node with itself, calculated as the trace of the adjacency matrix,
- cycles of two nodes (L2): sum of all loops containing two nodes, calculated by the trace of the squared adjacency matrix divided by two,
- cycles of three nodes (L3): sum of all loops containing three nodes (triangles), calculated by the trace of the cubed adjacency matrix divided by three.”

Global attributes:

- “average total degree (ATD): given a node n, the Total Degree is the sum of “in and out” edges. Average Total Degree is the sum of Total Degree of all nodes divided by the number of nodes,
- density: number of edges divided by possible edges. (D = E/N^2^), where E is the number of edges and N is the number of nodes,
- diameter: length of the longest shortest path between the node pairs of a network.
- average shortest path (ASP): average length of the shortest path between pairs of nodes of a network,
- clustering coefficient (CC): Given a node n, the Clustering Coefficient Map (CCMap) is the set of fractions of all n neighbours that are also neighbours of each other. Average CC is the sum of the Clustering Coefficients of all nodes in the CCMap divided by number of elements in the CCMap.”

### 2.4. Statistical Analysis

Demographic variables were compared with X2 and ANOVA tests (Table 1). One-way ANOVA with a factor of group (SZ, BD, and HC) with Tukey’s HSD post-hoc tests were used to compare SGA parameters and standard measures with normal distribution and equal variances. Standard measures and SGA parameters that did not meet assumptions for ANOVA were compared with the use of the Kruskal–Wallis test (Table 2 and Table 3). For the non-continuouss variables (RE, PE, L1, L2, L3, and CC) due to the expected values <5, Fisher’s tests were used. Series of logistic regressions adjusted for age and gender were used to evaluate associations between SGA parameters and diagnoses. The area under the receiver operating characteristic curve (AUC) was used to estimate classification quality of the above-mentioned variables between SZ, BD, and HC groups. Quality was considered excellent when AUC was higher than 0.8, good when AUC ranged from 0.6 to 0.8, and poor when AUC was smaller than 0.6.

## 3. Results

### 3.1. Standard Measures

Analysis of Phonemic and Semantic VFT with the use of standard measures revealed only two statistically significant differences. SZ patients showed lower word count (*p* = 0.01) and lower total number of correct words in Semantic VFT than the HC group (*p* = 0.006; Table 2 and Table 3). There were no statistically significant differences between BD and HC groups in terms of standard measures.

### 3.2. SGA Comparisons

Results of SGA analyses are presented in Table 2 and Table 3. SZ patients’ Semantic VFT graphs showed lower total word count (*p* = 0.01), less nodes (*p* = 0.003) and edges (*p* = 0.010), higher density (*p* = 0.005), and smaller diameter (*p* < 0.001) and average shortest path (*p* < 0.001) than the HC group.

BD patients’ Semantic VFT graphs presented smaller diameter (*p* = 0.015) and average shortest path (*p* = 0.024) than HC. Phonemic VFT SGA comparisons revealed no statistically significant differences between BD and HC groups.

There were no statistically significant differences between SZ and BD patients in terms of Phonemic and Semantic VFT SGA. Also, we have shown no significant differences between SZ, BD, and HC groups in terms of Phonemic VFT SGA.

### 3.3. Logistic Regression Analysis:

Decreased values of the following Semantic VFT parameters were associated with the higher probability of being in the SZ rather than HC group: N (OR = 0.86, *p* = 0.010), E (OR = 0.88, *p* = 0.020), WC (OR = 0.88, *p* < 0.05), Diameter (OR = 0.86, *p* = 0.001), ASP (OR = 0.63, *p* = 0.001).

An increased density of the Semantic VFT graph was associated with a higher probability of having a diagnosis of SZ, rather than being in HC group (OR = 1.02, *p* = 0.020).

Decreased Phonemic VFT graph diameter (OR = 0.85, *p* = 0.030) and ASP (OR = 0.61, *p* = 0.030) were associated with a higher probability of being in the SZ group, rather than the HC group.

None of the Semantic and Phonemic SGA allowed the determination of whether the participant was in the BD or HC group. Additionally, none of above-mentioned parameters allowed the prediction of whether the patient was in the BD or SZ group. A detailed description of logistic regression analysis is presented in Table 4.

ROC analysis has shown that following semantic verbal fluency parameters reached significant, good classification quality across SZ and HC groups: N (AUC = 0.73, *p* < 0.001), E (AUC = 0.71, *p* < 0.01), WC (AUC = 0.71), *p* < 0.001), Density (AUC = 0.73, *p* < 0.001), Diameter (AUC = 0.75, *p* < 0.001), and ASP (AUC = 0.75, *p* < 0.001). Two phonemic verbal fluency parameters showed significant, good classification quality across SZ and HC groups: Diameter (AUC = 0.68, *p* < 0.01) and ASP (AUC = 0.68, *p* < 0.01). Three semantic verbal fluence parameters showed significant, good classification quality across BD and HC groups: Density (AUC = 0.66, *p* < 0.05), Diameter (AUC = 0.68, *p* < 0.01), and ASP (AUC = 0.68, *p* < 0.01).

## 4. Discussion

To our best knowledge, this is the first study applying graph theory-based analysis theory to compare SZ, BD, and HC groups’ Semantic and Phonemic VFT performance. The comparison of three groups in terms of standard measures revealed differences in only two variables. SZ patients showed a decreased word count and lower total number of correct words in Semantic VFT than the HC group. An analysis of speech graphs revealed the presence of variables reflecting the complex disturbances of verbal fluency, differentiating patients and HC groups. We have also found that none of the Phonemic VFT parameters were able to differentiate BD, SZ, and HC groups.

According to our hypotheses, SZ patients’ Semantic VFT graphs presented a lower number of nodes, edges, and disturbances within global attributes: higher density, smaller diameter, and decreased ASP, compared to HC. A decreased number of nodes and edges corresponds directly to the lower word count, including the lower number of correct responses. Alterations of global attributes indicate that patients generated non-linear speech graphs, with shorter paths through the first word to the last one, and with additional, unnecessary connections between produced words. Speech graph analysis enabled us to demonstrate the presence of qualitative differences in Semantic VFT that would otherwise go unnoticed in the routine comparison of groups using standard measures. Our results are consistent with other studies using different analysis methods applied to the semantic networks produced by individuals with SZ, such as multidimensional scaling and clustering techniques [21,30,31]. These studies indicate the lack of organization, and the reduction in the size of lexicon in the patients group.

The mechanisms of semantic fluency disturbances in SZ are not clear. Data suggests that decreased production of category exemplars may represent an increase in time that it takes SZ patients to move among related nodes in a semantic network, which should be proximal in semantic space [9]. This may be associated with the failures in spreading activation across associative connections [9,30,32,33]. Also, it has been suggested that patients may produce fewer number of words because they may allocate more time to inhibit incorrect, or monitor otherwise problematic, responses [9,34]. Our results provide further support for the cognitive deficits of the semantic store in SZ. Interestingly, Bertola et al., 2014, have shown that the same SGA differentiated patients with mild cognitive impairment, Alzheimer’s disease, and the control group [5]. They have shown that, with a decreasing functional performance and cognitive impairment, Semantic VFT graphs became denser, with a smaller diameter, and an ASP with fewer numbers of nodes and edges [5]. Future studies should evaluate the association between semantic networks and cognitive functions and functional activity measures in SZ. We have shown that SZ patients present alterations within speech graphs derived from Semantic VFT and preserved Phonemic VFT networks, which supports observations of a disproportionate impairment of semantic verbal fluency in SZ [10]. A meta-analytic review of Henry and Crowford (2005) showed that SZ patients were more impaired in semantic relative to phonemic verbal fluency, suggesting that, in addition to general retrieval difficulties, SZ is associated with compromises to the semantic store [35].

Unlike standard measures, SGA analysis showed differences between BD and HC groups. BD patients differed significantly in the diameter and ASP of the speech graphs obtained from Semantic VFT. Thus, they produced non-linear, less direct, and poorer networks with significantly smaller diameters. Though there were no significant differences between SZ and BD performance, a smaller number of SGA was able to distinguish the BD group from HC compared to SZ. This may be associated with the observation that, in our study, contrary to the SZ group, BD patients did not differ from the HC group in terms of standard measures. Despite that, SGA made it possible to distinguish between the two groups, suggesting that, in the case of BD patients, speech graph analysis may be more sensitive in differentiating both groups than comparisons based solely on productivity and error scores. Diameter and ASP values were also shown to be the most prevalent differences across the healthy elderly, mild cognitive impairment group, and the Alzheimer’s disease group [5], and may be the most defined characteristic associated with general cognitive deficits. It has been shown that working memory, executive functioning, and processing speed score are related to the Semantic and Phonemic VFT output of BD patients [36]. We encourage future studies to use SGA in order to evaluate associations between verbal fluency and other cognitive domains in BD. Contrary to results of the recent meta-analysis, we have shown no differences in terms on Phonemic VFT between BD and HC groups [12]. Semantic VFT scores may present greater sensitivity to the cognitive impairment in the semantic memory, organization, and retrieval [35]. Given the fact that, in our study, BD patients did not differ in terms of standard measures with HC, our results suggest that semantic VFT scores may be more sensitive than phonemic VFT to spot verbal fluency deficits in the group of relatively well performing BD individuals.

In our study, SZ and BD patients did not present significant alterations of the recurrence attributes, e.g., repeated or parallel edges, and cycles between nodes. The recurrence attributes are related to the number of repetitions during VFT, which did not distinguish between the three examined groups. Surprisingly, this simple standard measure of repetitions is rarely analyzed in the literature. In the recent meta-analysis of SZ VFT done by Tan et al., 2020, the perseverative/repeat scores were reported only in 9 out of 48 studies [11]. The number of repetitions did not differentiate healthy controls from patients with chronic SZ [23,37,38,39]. Galaverna et al., 2014, showed a higher number of perseverative errors in patients with chronic SZ [40]. In contrast, Kosmidis et al., 2005, showed a lower number of repetitions in the SZ group. Interestingly, in the BD literature, the types of errors during VFT are not evaluated [41]. In a recent meta-analysis performed by Rauchere-Chene et al., 2016, perseverative errors were not analyzed [12]. Bertola et al., 2014, comparing patients with mild cognitive impairment and Alzheimer’s disease, showed that even the groups that did not differ in the number of perseverations, did differ in the occurrence of loops of three nodes [5]. This suggests that speech graphs’ recurrence attributes may constitute a more sensitive measure of perseverative measures than a total number of repetitions. It was suggested that the impairments in the central executive and episodic buffer functions of working memory could explain the repetition of words during VFT [5]. Given the fact that both BD and SZ patients show deficits of executive functions, including working memory impairments [8], we suggest using speech graphs’ recurrence attributes to measure perseverations during verbal fluency tasks in these disorders.

None of the standard measures and SGA analyzed in this study were able to differentiate BD and SZ patients. The absence of differences between the groups, established by the new graph theory-based approach, corroborates the results of a qualitative review and recent studies indicating a comparable level of verbal fluency impairments in both clinical groups [14,15,16,17,18]. Our results stay in line with the “schizophrenia-bipolar disorder boundary” hypothesis, suggesting that the verbal fluency deficits may present one of the intermediate phenotypes in both SZ and BD [1].

We are aware of the limitations of our study, such as relatively small groups, the fact that groups of patients were not drug-naïve, and our lack of ability to evaluate potential effects of medication on VFT results due to the small subject sample. To eliminate a possible bias of our findings related to antipsychotic treatment, we only recruited patients taking antipsychotic drugs from the group of dibenzoxazepines, and therefore provided a relative pharmacological homogeneity across patient groups.

## 5. Conclusions

In conclusion, our study, for the first time, compared BD, SZ, and HC in terms of VFT performance with the novel graph analysis approach. The application of this method revealed that, in the SZ group, semantic verbal fluency deficits are not only limited to the productivity scores. Our study showed a complex picture of semantic network disturbances in this clinical group, indicating that SZ patients’ speech graphs present a higher density; smaller diameter, ASP, and LSC; as well as less nodes and edges compared to HC. In the case of BD patients, SGA were able to distinguish patients from HC, despite there being a lack of differences between the two groups in standard measures. Interestingly, none of the variables characterizing verbal fluency performance were able to differentiate SZ and BD. Our results encourage the use of speech graph analysis, as it reveals verbal fluency alterations that remained unnoticed in the routine comparisons of groups with the use standard measures. As these additional calculations are no extra burden on patients’ time, future studies regarding verbal fluency deficits in SZ and BD should include the evaluation of SGA, in order to better understand this issue.

## Figures and Tables

**Table 1:** The description of study groups. BD—bipolar disorder, SZ—schizophrenia, HC—healthy controls.

	BD	SZ	HC
Age ^a^ (mean years ± SD)	36.31 ± 11.63	35.37 ± 11.40	39.04 ± 14.11
Sex ^b^ (men/women)	11/18	12/17	15/14
Antipsychotic medication (number of patients, %)			
Olanzapine	7 (24%)	20 (68%)	
Quetiapine	16 (55%)	2 (6%)	
Clozapine	1 (3%)	6 (21%)	
Normothymic (number of patients, %)			
Valproic acid	14 (48%)		
Lamotrigine	2 (7%)		

^a^—ANOVA, F(2, 84), *p* = 0.511, ^b^—X2(2, 84) = 0.66, *p* = 0.72.

**Table 2:** Comparison of semantic fluency test measures between schizophrenia, bipolar disorder, and healthy controls group. SZ—schizophrenia; BD—bipolar disorder; HC—healthy controls; WC—word count; N—nodes; RE—repeated; PE—parallel edges; L1, L2, L3—cycles of one, two, or three nodes; LSC—largest strongly connected component; ATD—average total degree; ASP—average shortest path; CC—clustering coefficient; SD—standard deviation. ^a^—ANOVA; ^b^—Kruskal–Wallis test; ^c^—Fisher’s test.

	Mean Score (SD)			Df	Test’s Statistics (F or H)	SZ vs. HC	BD vs. HC	SZ vs. BD
	SZ Group	BD Group	HC Group					
Semantic Fluency Test								
Standard measures								
WC ^a^	19.83 (5.54)	21.45 (7.74)	25.07 (7.42)	2, 84	4.30 *	*p* = 0.015	ns.	ns.
Total no. of correct words ^a^	18.83 (5.39)	21.00 (6.31)	24.66 (7.57)	2, 84	5.98 **	*p* = 0.003	ns.	ns.
Total no. of errors ^b^	0.03 (0.186)	0 (0)	0.03 (0.186)	2	1.012	-	-	-
Total no. of repetitions ^b^	0.66 (1.08)	0.83 (1.28)	0.72 (0.96)	2	0.606	-	-	-
Speech Graph Attributes								
N ^a^	18.83 (5.39)	21.00 (6.31)	24.66 (7.57)	2, 84	5.98 **	*p* = 0.003	ns.	ns.
E ^a^	18.86 (5.51)	21.14 (6.78)	24.07 (7.42)	2, 84	4.51 **	*p* = 0.010	ns.	ns.
RE ^c^	0.03 (0.19)	0.03 (0.19)	0 (0)	2, 84	1.27	-	-	-
LSC ^b^	5.45 (6.80)	5.38 (5.86)	4.03 (5.30)	2	1.80	-	-	-
PE ^c^	0.10 (0.31)	0.14 (0.581	0.03 (0.19)	-	1.11	-	-	-
L1 ^c^	0.03 (0.19)	0.21 (0.62)	0 (0)	-	4.48	-	-	-
L2 ^c^	0.07 (0.26)	0.10 (0.409	0.03 (0.19)	-	0.61	-	-	-
L3 ^c^	0.03 (0.19)	0.14 (0.35)	0 (0)	-	4.48	-	-	-
ATD ^b^	2.01 (0.17)	2.01 (0.17)	1.95 (0.07)	2	0.78	-	-	-
Density ^a^	0.12 (0.04)	0.11 (0.04)	0.09 (0.04)	2, 84	10.13 **	*p* = 0.005	ns.	ns.
Diameter ^a^	14.34 (6.14)	16.59 (7.15)	21.76 (8.53)	2, 84	7.78 ***	*p* < 0.001	*p* = 0.02	ns.
ASP ^a^	5.59 (1.96)	6.28 (2.31)	7.97 (2.82)	2, 84	7.58 ***	*p* < 0.001	*p* = 0.02	ns.
CC ^c^	0.01 (0.03)	0.02 (0.05)	0 (0)	-	4.32	-	-	-

*—*p* ≤ 0.05, **—*p* ≤ 0.01, ***—*p* ≤ 0.001.

**Table 3:** Comparison of phonemic fluency test measures between schizophrenia, bipolar disorder, and healthy controls group. SZ—schizophrenia; BD—bipolar disorder; HC—healthy controls; WC—word count; N—nodes; RE—repeated; PE—parallel edges; L1, L2, L3—cycles of one, two, or three nodes; LSC—largest strongly connected component; ATD—average total degree; ASP—average shortest path; CC—clustering coefficient; SD—standard deviation. ^a^—ANOVA; ^b^—Kruskal–Wallis test; ^c^—Fisher’s test.

	Mean Score (SD)			Df	Test’s Statistics (F or H)	SZ vs. HC	BD vs. HC	SZ vs. BD
	SZ Group	BD Group	HC Group					
Phonemic Fluency Test								
Standard measures								
WC ^a^	16.03 (5.03)	17.52 (5.76)	17.52 (5.44)	2, 84	0.72	-	-	-
Total no. of correct words ^a^	15.34 (4.84)	16.93 (5.43)	17.07 (5.18)	2, 84	1	-	-	-
Total no. of errors ^b^	0.14 (0.44)	0.59 (1.52)	0.17 (0.47)	2	1.552	-	-	-
Total no. of repetitions ^b^	0.79 (0.98)	0.48 (0.79)	0.41 (0.78)	2	3.661	-	-	-
Speech Graph Attributes								
N ^a^	15.34 (4.84)	16.93 (5.43)	17.07 (5.18)	2, 84	1	-	-	-
E ^a^	15.03 (5.03)	16.55 (5.83)	16.52 (5.44)	2, 84	0.73	-	-	-
RE ^c^	0 (0)	0 (0)	0.03 (0.19)	-	0.43			
LSC ^b^	5.24 (4.96)	3.97 (5.86)	3.45 (4.74)	2	4.32	-	-	-
PE ^c^	0.03 (0.19)	0.03 (0.19)	0.03 (0.19)	-	0.43	-	-	-
L1 ^c^	0 (0)	0.03 (0.19)	0.03 (0.19)	-	1.27	-	-	-
L2 ^c^	0.03 (0.19)	0.03 (0.19)	0 (0)	-	1.27	-	-	-
L3 ^c^	0.07 (0.26)	0.03 (0.19)	0.03 (0.19)	-	0.69	-	-	-
ATD ^b^	1.94 (0.13)	1.93 (0.15)	1.93 (0.23)	2	1.05	-	-	-
Density ^b^	0.16 (0.08)	0.14 (0.07)	0.14 (0.11)	2	2.56	-	-	-
Diameter ^a^	11.24 (4.52)	13.39 (5.56)	14.48 (5.38)	2, 84	2.94	-	-	-
ASP ^a^	4.51 (1.48)	5.15 (2.04)	5.54 (1.77)	2, 84	2.49	-	-	-
CC ^c^	0.01 (0.04)	0.01 (0.02)	0 (0.02)	-	0.69	-	-	-

**Table 4:** Logistic regression analyses evaluating association between verbal fluency variables and the diagnosis. SZ—schizophrenia; BD—bipolar disorder; HC—healthy controls; WC—word count; N—nodes; RE—repeated; PE—parallel edges; L1, L2, L3—cycles of one, two, or three nodes; LSC—largest strongly connected component; ATD—average total degree; ASP—average shortest path; CC—clustering coefficient.

	OR	−95% CI	+95% CI	R2 (Nagelkerke)	*p*
Semantic Fluency Test					
N					
HC vs. SZ	0.86 **	0.79	0.95	0.26	0.01
E					
HC vs. SZ	0.88 **	0.81	0.97	0.21	0.02
WC					
HC vs. SZ	0.88 **	0.81	0.97	0.21	0.02
Density					
HC vs. SZ	1.02 **	1.004	1.04	0.2	< 0.001
Diameter					
HC vs. SZ	0.86 ***	0.78	0.94	0.33	0.001
HC vs. BD	0.92 *	0.86	0.99	0.16	-
ASP					
HC vs. SZ	0.63 ***	0.48	0.83	0.32	0.001
HC vs. BD	0.78 *	0.62	0.97	0.15	-
Phonemic fluency test					
Diameter					
HC vs. SZ	0.85 **	0.75	0.96	0.20	0.03
ASP					
HC vs. SZ	0.61 **	0.42	0.90	0.19	0.03

*—*p* ≤ 0.05, **—*p* ≤ 0.01, ***—*p* ≤ 0.001. Only statistically significant results are presented in the table.

## Data Availability

Data is available on request.
